# Insulin Signaling Mediates Sexual Attractiveness in *Drosophila*

**DOI:** 10.1371/journal.pgen.1002684

**Published:** 2012-04-26

**Authors:** Tsung-Han Kuo, Tatyana Y. Fedina, Ingrid Hansen, Klaus Dreisewerd, Herman A. Dierick, Joanne Y. Yew, Scott D. Pletcher

**Affiliations:** 1 Huffington Center on Aging, Baylor College of Medicine, Houston, Texas, United States of America; 2 Department of Molecular and Human Genetics, Baylor College of Medicine, Houston, Texas, United States of America; 3 Department of Molecular and Integrative Physiology and Geriatrics Center, University of Michigan, Ann Arbor, Michigan, United States of America; 4 Program in Cell and Molecular Biology, Baylor College of Medicine, Houston, Texas, United States of America; 5 Institute of Medical Physics and Biophysics, University of Münster, Münster, Germany; 6 Temasek Life Sciences Laboratory, National University of Singapore, Singapore, Singapore; 7 Department of Biological Sciences, National University of Singapore, Singapore, Singapore; University of California Davis, United States of America

## Abstract

Sexually attractive characteristics are often thought to reflect an individual's condition or reproductive potential, but the underlying molecular mechanisms through which they do so are generally unknown. Insulin/insulin-like growth factor signaling (IIS) is known to modulate aging, reproduction, and stress resistance in several species and to contribute to variability of these traits in natural populations. Here we show that IIS determines sexual attractiveness in *Drosophila* through transcriptional regulation of genes involved in the production of cuticular hydrocarbons (CHC), many of which function as pheromones. Using traditional gas chromatography/mass spectrometry (GC/MS) together with newly introduced laser desorption/ionization orthogonal time-of-flight mass spectrometry (LDI-MS) we establish that CHC profiles are significantly affected by genetic manipulations that target IIS. Manipulations that reduce IIS also reduce attractiveness, while females with increased IIS are significantly more attractive than wild-type animals. IIS effects on attractiveness are mediated by changes in CHC profiles. Insulin signaling influences CHC through pathways that are likely independent of dFOXO and that may involve the nutrient-sensing Target of Rapamycin (TOR) pathway. These results suggest that the activity of conserved molecular regulators of longevity and reproductive output may manifest in different species as external characteristics that are perceived as honest indicators of fitness potential.

## Author Summary

In nature, a myriad of specialized traits have evolved that are used for intraspecific communication and mate choice. We postulated that certain traits may have evolved to be attractive by virtue of their accurate representation of molecular pathways that are critical for determining evolutionary fitness. Insulin signaling (IIS) is one such pathway. It has been shown to modulate aging, reproduction, and stress resistance in several species and to contribute to variability of these traits in natural populations. We therefore asked whether IIS affected key sexual characteristics and overall attractiveness in the fruit fly *Drosophila melanogaster*. We found that IIS regulates cuticular hydrocarbons (the key pheromones in flies), that reduced IIS also reduced attractiveness, and that flies with increased IIS were significantly more attractive than wild-type animals. Further experiments revealed that these effects may also be influenced by a second conserved nutrient-sensitive pathway, the TOR pathway. We suggest that natural selection may have favored a plethora of species-specific sexual characteristics because they accurately represent a small number of influential pathways that determine longevity and reproductive output across taxa. In other words, it may be that, whether fly or human, beauty is more than skin-deep.

## Introduction

Organismal fitness is influenced by social interactions, which drive sexual selection and individual attractiveness. In nature, a myriad of specialized signals and cues are used for intraspecific communication and mate choice, and many attractiveness traits are known to reflect an individual's health and reproductive value. These indicator traits are presumed to be reliable because they are either costly to produce/maintain and therefore difficult to fake [1] or because they are subject to direct physiological constraints [2]. Regardless of their nature, effective quality indicators must be an honest reflection of an individual's reproductive potential [3], [4] and as such, must be linked at the molecular level to the key fitness parameters —longevity and reproductive output— that they represent. However, very few studies have identified specific molecular relationships that link attractive traits to the pathways that influence overall health and individual fitness (reviewed in [5]).

In *Drosophila melanogaster*, attractive traits include cuticular hydrocarbons (CHC), which are long-chain lipids deposited on the insect cuticle [6]. Their presumed ancestral function is desiccation resistance [7], but they also play major roles in insect social communication, species recognition, and as sex pheromones [8]–[10]. In *Drosophila*, manipulation of certain neuropeptide and endocrine systems, such as dopamine or juvenile hormone [11], affect CHC profiles, but the biological function of these alterations in CHC are unclear. At the molecular level, several genes have been implicated in CHC synthesis [12]–[15], but there is little information about the mechanisms that regulate their expression.

Insulin/insulin-like growth factor signaling (IIS) is an evolutionarily conserved pathway that influences animal development, metabolism, longevity, and fecundity [16], [17]. Reduced IIS generally extends lifespan, but it is normally accompanied by reduced reproduction [18], [19]. Conversely, increasing insulin signaling results in increased body weight and fecundity [20]. Pleiotropic effects like these are not uncommon, and they likely represent underlying trade-offs associated with the plasticity through which organisms alter their life history characteristics in response to environmental conditions to maximize individual fitness [16], [18], [19]. For example, animals with reduced insulin signaling are more likely to survive periods of acute stress or prolonged malnutrition, but they are readily outcompeted when nutrients are replete [21]. Standing genetic variation is also known to influence basal transcript levels of IIS pathway genes in flies [22], [23] and in humans [24] with potentially long-term effects on phenotypic condition (e.g. obesity in humans, [25]), and developmentally-determined traits (e.g. beetle horns, [26]). IIS is therefore likely to be an important mechanism through which many different organisms respond to variable environmental conditions to maximize fitness [21].

We hypothesized that certain attractive traits might represent conspicuous extensions of molecular pathways that are critical for determining fitness. Because fitness components are strongly influenced by shifts in resource allocation in response to changing environmental conditions, we reasoned that the chooser/assessor will be most interested in the immediate physiological state of a potential mate and that relevant pathways are likely to be master regulators of resource allocation. The IIS pathway was an obvious candidate to test.

## Results/Discussion

### Genetic manipulation of insulin signaling significantly alters CHC profiles

To test our hypothesis we focused our initial experiments on CHC profiles in female flies carrying a loss of function mutation in the insulin receptor substrate, *chico*. *chico* mutant females have attenuated insulin signaling, and they are small, long-lived, and sterile [27]. We reasoned that studying female profiles would provide a clearer picture of the links between IIS and attractiveness because female attractiveness, unlike male, is less influenced by behavior and because the effects of IIS manipulation on lifespan and reproductive output are better understood, phenotypically and genetically, in females [28], [29]. In nature, male choice is important in many species [30], [31], including *Drosophila*, where mating opportunities are constrained by allocation of time and energy into courtship and ejaculate production [32].

**Figure 1:**
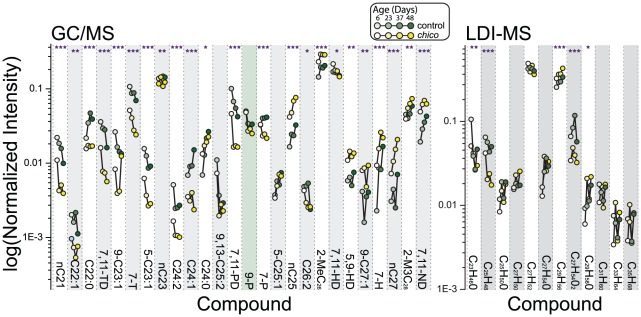
Alterations in insulin signaling impact female CHC profiles. Using both GC/MS and LDI-MS analysis, we found dramatic differences in female CHC profiles caused by mutation of the insulin-receptor substrate *chico*, which results in a reduction in insulin signaling [27]. Data points representing age-dependent measures of the same compound are connected by lines; *chico* mutant data are plotted in yellow and those of control in green. Ages of the flies at the time of measurement (6, 23, 37, and 48 days) are further distinguished by shading. Error bars are omitted for clarity. A few compounds are identified by both methods: C_25_H_48_ (LDI-MS) corresponds to 9,13-C25:2 and 7,11-PD (GC/MS); C_27_H_52_ to 7,11-HD and 5,9-HD; and C_29_H_56_ to 7,11-ND. Significant changes with aging are not indicated but are similar to published data [36]. P-values reflect differences in individual compounds between *chico* and control flies determined by the genotype main effect in a two-factor ANOVA. *P<0.05, **P<0.01, ***P<0.001.

Gas chromatography/mass spectrometry (GC/MS) and laser desorption/ionization orthogonal time-of-flight mass spectrometry (LDI-MS) were used to generate comprehensive CHC profiles in *chico* mutant and control flies sampled at four different ages (6, 23, 37 and 48 days post-eclosion) [33]–[35]. *chico* flies exhibited significant differences in the levels of most compounds (23/26 compounds in the GC/MS and 5/12 compounds in the LDI-MS analysis) (Figure 1). The number of differences was substantially greater than the expected number based on chance alone (1.3 differences for α = 0.05). Furthermore, of the 23 differences that were significant based on individual tests, 20 remained significant after a Holm-Bonferroni correction for multiple testing (7,11-heptacosadiene [7,11-HD], C26:2, and C24:0 did not achieve the modified threshold). Age-dependent changes in CHC profiles corresponded well with previous studies [36], and we were surprised to observe that these patterns were largely unaffected by *chico* mutation, despite a significant extension of their lifespan [27]. Only one CHC exhibited a statistically significant interaction between genotype and age (7-heptacosene, 7-H), suggesting that the majority of age-dependent CHC changes were independent of the mutation in *chico* (Figure 1).

To confirm that the observed phenotypes in *chico* mutants were indeed due to modulation of IIS, we measured changes in CHC profiles following manipulation of other components of the pathway. InR is the single insulin receptor in *Drosophila*, which binds insulin-like peptides and leads to activation of Akt kinase [37]. Pten phosphatase antagonizes IIS [38]. To avoid the developmental consequences associated with IIS manipulation, we employed the Geneswitch system (driven by a ubiquitous tubulin promoter in response to the drug RU486) together with *UAS-Akt^RNAi^, UAS-Pten, or UAS-InR* to target transgene expression to adult flies. Comparisons were then made between adult females that experienced transgene expression following exposure to RU486 and control animals of the same genotype that were not exposed to the drug.

Down-regulation of IIS through expression of *UAS-Akt^RNAi^* or *UAS-Pten* phenocopied the effects of *chico* mutation. Changes in CHC caused by the *chico* mutation and the two transgenic manipulations were highly positively correlated (Figure S1), and consistent changes were observed for several individual CHC. We observed reductions of 7-tricosene (7-T), *n*-tricosane (nC23), 9-pentacosene (9-P), 7,11-pentacosadiene (7,11-PD in GC/MS and C_25_H_48_ in LDI-MS), and 7-pentacosene (7-P). The levels of 2-methylhexacosane (2-MeC_26_), 5,9-heptacosadiene (5,9-HD) and 7,11-nonacosadiene (7,11-ND in GC/MS and C_29_H_56_ in LDI-MS) were increased (Figure S2, Table S1).

Activation of IIS through overexpression of *InR* produced effects on CHC profiles that were generally the converse of those generated by IIS knock-down. There was a highly significant negative correlation between CHC changes in *chico* mutant flies and *InR* over-expressing animals (Figure S1C), with overexpressing females exhibiting greater levels of 7-T, 9-P, 7,11-PD, and 7-P and reduced levels of 2-MeC_26_, 5,9-HD and 7,11-ND (Figure S2, Table S1). We note that RU486 alone had no significant effects on CHC profiles (Figure S3A). Together these data show that modulation of IIS is capable of both increasing and decreasing the representation of specific CHC from the levels observed in wild-type animals.

### Insulin signaling promotes attractiveness

**Figure 2:**
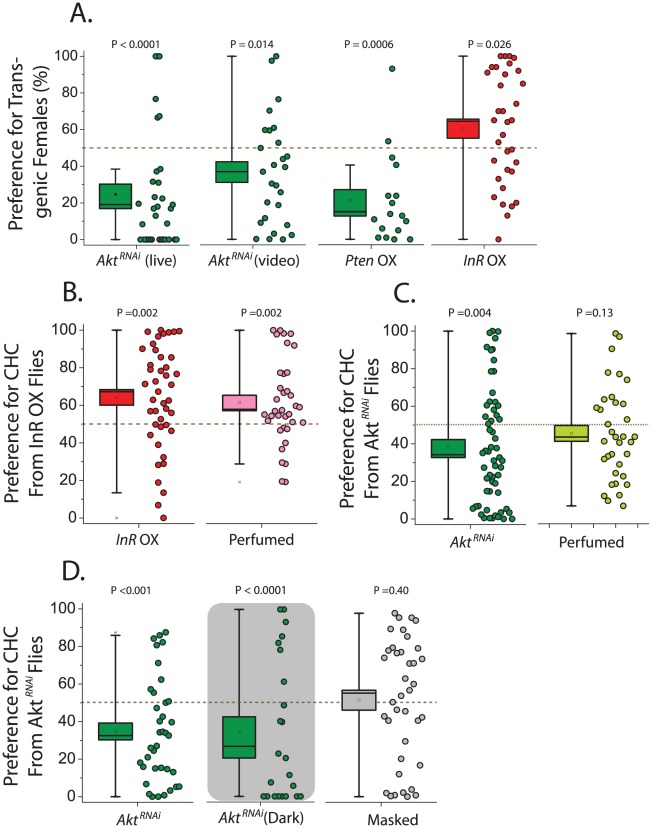
Female attractiveness is influenced by the insulin signaling pathway. (A) Males prefer control females compared to *Akt* knockdown females, as was determined using both live courtship assay (N = 33) and video analysis (N = 27). Overexpression (OX) of *Pten* also decreases female attractiveness (N = 27), while overexpression of *InR* increases attractiveness (N = 34). (B) In separate trials, male preference for *InR* overexpressing flies remained strong (N = 43), and they also exhibited significance preference for oenocyte-less females that were perfumed with CHC from flies overexpressing *InR* compared to oenocyte-less females perfumed with CHC from control females (N = 36). (C) Also in separate trials, males exhibited decreased preference for flies expressing *Akt^RNAi^* (N = 59), and a strong trend for decreased preference of their corresponding CHC was observed when oenocyte-less females were perfumed (N = 35). (D) Loss of attractiveness with knockdown of *Akt* persists in the dark (live assay; N = 23), and this male preference was lost following a ‘masking’ procedure whereby transgenic females were perfumed with CHC from wild-type Canton-S females (N = 38). For all behavioral assays, preference is presented as the percentage of time choosing animals spend either actively courting (live analysis) or in the courtship radius of (video analysis) the mutant fly divided by the total time attributed to both test flies. P values represent either the results of a Wilcoxon signed-rank test applied to test the null hypothesis of no preference (no difference from 50%) or the results of a bootstrap randomization procedure (see Materials and Methods). Box boundaries reflect the SEM and whiskers delineate the 80^th^ and 20^th^ percentiles of the data.

Having established that alterations in IIS impact CHC profiles, we next asked whether these changes affect sexual attractiveness. *chico* mutant flies were not studied in this context because of their small size [39]. We instead began by examining female attractiveness in *Akt* knockdown flies by assessing male preference to decapitated females in a two-choice courtship assay using live observation and video tracking. We found that wild-type Canton-S males spend significantly less courtship time with GeneSwitch>*UAS-Akt^RNAi^* females exposed to RU486 (thus expressing the RNAi) compared to females not exposed to the drug (Figure 2A). Inhibition of IIS by overexpression of *Pten* also decreased female attractiveness, while activation of the pathway through *InR* overexpression increased attractiveness (Figure 2A). Control animals in these experiments are genetically identical but have not been exposed to the drug RU486, which induces transgene expression and itself has no effect on attractiveness (Figure S3C).

To confirm that preferences were based on chemical cues, CHC transfer experiments were conducted. We “perfumed” same-age oenocyte-less flies, which do not produce CHC [40], with either CHC from control flies or flies in which IIS was manipulated. We then tested male preference and found that males preferred oenocyte-less females perfumed with CHC from animals that overexpress *InR* over those covered with CHC from their corresponding control animals (Figure 2B). By design all characteristics except transferred CHC were effectively identical between perfumed oenocyteless females, demonstrating that CHCs are responsible for IIS-dependent increases in female attractiveness in our 2-choice assays. Conversely, experiments using UAS-*Akt^RNAi^* resulted in reduced preference for oenocyte-less flies perfumed with CHC from *Akt* knockdown animals compared to CHC drawn from their controls (Figure 2C). The *Akt^RNAi^* perfuming results were consistently more variable than those obtained using transgenic animals directly, and a strong trend was consistently observed (Figure 2C). However, when courtship assays were performed in the dark to exclude potential involvement of visual cues, a strong preference for control females remained, and when Geneswitch - UAS-*Akt^RNAi^* transgenic animals either fed or not fed RU486 were perfumed with CHC from wild-type Canton-S females, their differences in attractiveness were masked (Figure 2D). These data further support the notion that differences in CHC are responsible for the differences in attractiveness. Consistent with its effects on female CHC profiles, therefore, modulation of the IIS pathway can both increase or decrease the attractiveness of wild-type females.

Our data reveal unexpected complexities by which individual CHC affect attractiveness. Several compounds are known to stimulate male courtship behaviors, including 7-P, 9-P, 7,11-HD and 7,11-ND [6], . While 7-P and 9-P levels were decreased following reduction in IIS, which is consistent with their reduced attractiveness, we did not observe significant changes in the levels of 7,11-HD. More surprising was that 7,11-ND, which is thought to promote male courtship, was increased following reduced IIS. Incidentally, an increase in 7,11-ND levels was recently observed in aging flies, which also resulted in reduced attractiveness [36]. It is possible that potent and unidentified pheromones are playing a large role in our observed effects. Candidates include C_27_H_54_O_2_, which is strongly promoted by IIS, and 2-MeC_26_, which is reduced.

**Figure 3:**
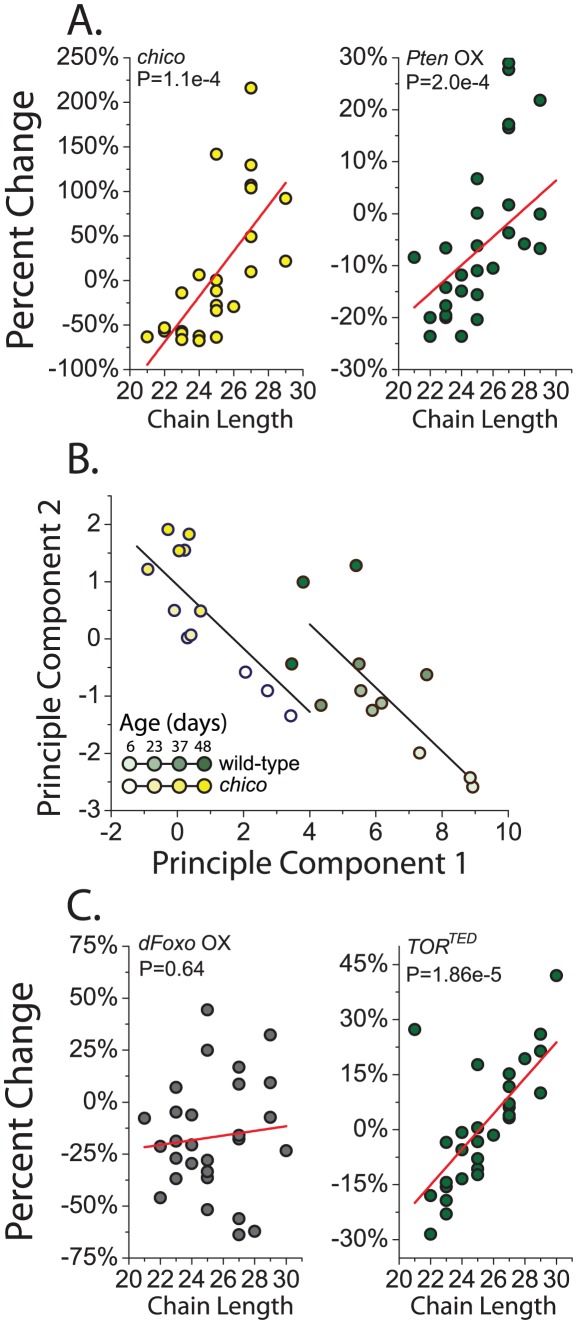
Reduced insulin signaling results in an increased proportion of longer chain CHC. (A) Knockout of *chico* and overexpression of *Pten* (*Pten OX*) decrease the amount of CHC with shorter chain lengths expressed on female flies and simultaneously increase the levels of compounds with longer chain lengths. Each data point represents the percentage change of normalized CHC expression level in *chico* mutant flies from their controls for a single compound from GC/MS. *chico* effects were calculated based on the genotype main effect across all measured ages (6, 23, 37, and 48 days old). (B) CHC profiles in young *chico* females resemble those of old control flies and they are affected similarly by aging. CHC profiles for control and *chico* mutant flies are represented by their first two principle components (see Materials and Methods). Each point represents a single sample in GC/MS data. ANCOVA analysis confirmed a significant effect of genotype on CHC profiles represented by each principal component, but the interaction term was not significant, suggesting that aging impacts components from each genotype similarly. Lines represent the fitted linear estimates from the ANCOVA. (C) Overexpression of *dfoxo (foxo OX)* failed to result in a relationship between the changes in specific compounds and their chain length. However, overexpression of the dominant negative *TOR^TED^* gene results in an increase in CHC with longer chain length. P-values are determined by regression analysis. *Pten*, *dfoxo*, and *TOR^TED^* flies were two weeks old when used for this analysis.

Attractiveness may instead be determined by global properties of CHC profiles rather than by the additive contribution of select compounds. *chico* mutant flies and flies overexpressing *Pten* had relatively more CHC with longer carbon chains and fewer CHC with shorter chain lengths (Figure 3A). Expression of *Akt^RNAi^* produced similar changes (P = 0.08, data not presented). RU486 alone had no systematic effect on CHC profiles of a control strain (Figure S3B). Aging has also been reported to result in increased longer-chained CHC [36], and the recurring similarities between reduced IIS and aging led us to examine this relationship more closely. Principle component analysis was used to distill changes in CHC across the profile into a small set of uncorrelated components and visually summarize their relationships. Based on the first two principle components (accounting for 57% of the variation), CHC profiles of young *chico* mutant flies resembled those of old control flies (Figure 3B). Aging impacted the components equally in both genotypes. Therefore, aging and IIS appear to act in parallel to shift the distribution of CHC in favor of those with longer carbon chains, which may reduce attractiveness. The similarities between young *chico* females and old control females may be reflective of their reduced reproduction.

### Insulin signaling regulates the expression of CHC synthesis enzymes

**Figure 4:**
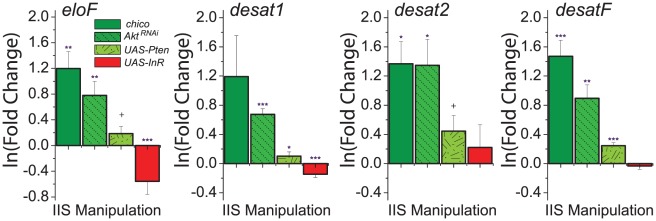
Genes involved in CHC synthesis are transcriptionally regulated by insulin signaling. Knock-down of insulin signaling by (*i*) loss of *chico*, (*ii*) knockdown of *Akt*, and (*iii*) overexpression of *Pten* increases the expression of enzymes known to be involved in CHC synthesis, including *eloF*, *desat1*, *desat2*, and *desatF*. Overexpression of *InR*, which enhances insulin signaling, decreases the expression of *eloF* and *desat1*. Expression levels are presented as the fold-change of the relevant genetic manipulation to control on the natural log scale. Error bars illustrate variability in biological replicates. P-values were determined by z-test applied to the null hypothesis of no change: ^+^P<0.1; *P<0.05, **P<0.01, ***P<0.001.

To explore the molecular mechanisms through which IIS modulates CHC profiles, we measured expression of genes involved in CHC synthesis. Given that reduced IIS increased the representation of longer-chained CHC (Figure 3A), we predicted that these manipulations would result in increased expression of *eloF*, which is female-specific and involved in long-chain hydrocarbon synthesis [13]. Indeed, we found that mRNA levels of *eloF* were significantly elevated in manipulations that reduced IIS, including *chico* mutation, *Akt^RNAi^*, and overexpression of *Pten* (Figure 4). Overexpression of *InR* had the opposite effect. Expression of *desat2*, which acts to produce 5,9-dienes, was significantly increased by reduction of IIS. Similar trends were observed for expression of *desat1*, which is required for the production of many alkenes [14], [43], and *desatF*, which introduces a second double bond to form female-specific dienes [12]. Together, these data suggest that IIS modulates CHC profiles at least in part through transcriptional regulation of the genes involved in their synthesis.

### Modulation of CHC and attractiveness is independent of dFOXO and may involve TOR signaling

Four lines of evidence suggest that the effects of IIS on CHC expression are largely independent of its canonical transcription factor target, *dfoxo*. First, reduced IIS leads to activation of dFOXO, but overexpression of *dfoxo* had a negligible effect on overall CHC profiles (Table S1). Second, there was no significant correlation between changes observed in *chico* mutant flies and flies overexpressing *dfoxo* (Figure S1D). Third, unlike all of our other IIS manipulations, there was no effect of *dfoxo* overexpression on compound chain length (Figure 3C). Forth, CHC regulatory gene expression changes that were observed in *chico* mutant animals largely persisted in *chico*; *dfoxo^w24^* double mutants (Figure S4).

Because of its emerging importance in the biology of aging we asked whether the modulation of the target of rapamycin (TOR) pathway might be involved in the effects of IIS on CHC profiles. These two pathways are known to interact. Many studies have implicated insulin signaling as an important regulator of TOR activity [44], [45], and TOR signaling can activate IIS intracellularly through phosphorylation of *Akt* [46]. We found that suppression of TOR signaling through transgenic overexpression of a dominant negative TOR (*UAS-TOR^TED^*) [47] resulted in CHC changes that were strongly positively correlated, but smaller in magnitude, to those induced by *chico* mutation (Figure S1E). There was also a significant effect of down-regulation of TOR signaling on the relative levels of CHC with greater chain length (Figure 3C). Together, our data suggest the hypothesis that alterations in IIS affect pheromone production and sexual attractiveness through mechanisms that are independent of *dfoxo* but involve the nutrient-sensing TOR pathway. Future studies focusing on specific TOR pathway modulators, such as S6K or 4E-BP, will be insightful in this regard. Finally, it may be interesting to examine the effect of juvenile hormone, which has been shown to influence fly CHC and is regulated by the IIS and TOR pathways, as potentially involved in the preferences that we report [11]. It has been linked to sexual attractiveness in other insect species.

### Conserved regulation of attractiveness: Is beauty more than skin-deep?

We have found that key attractive traits in *Drosophila melanogaster* females, specifically cuticular pheromones (a.k.a., cuticular hydrocarbons, or CHC), along with gene expression of CHC synthesis enzymes and attractiveness of females, robustly respond to genetic manipulations of the IIS pathway. Based on these data, we suggest that CHC are readily detectable manifestations of IIS pathway activity and that they are used as agents of choice because they provide individuals with information about the reproductive potential—in accordance with environmental conditions—of a possible mate.

Why might CHC profiles be the indicators of IIS activity in flies? A putative ancestral function of CHC in insects is prevention of water loss and resistance to desiccation. Flies may actively increase CHC production, specifically heavy-chain CHC, to protect against stressful environments, as in the case of reduced IIS. Alternatively, it may be that alterations in CHC are pleiotropic side-effects of IIS targeted to other physiological traits. For example, IIS may regulate triglyceride levels by modulating the expression of *desat1*, which has an important function in lipid metabolism [48]. Functions for *desat2* in starvation, cold resistance, and desiccation resistance have also been suggested [49]. Recent work has also shown that IIS influences female remating rate through unknown mechanisms likely related to metabolism, suggesting an additional link between this pathway and individual fitness [50].

**Figure 5:**
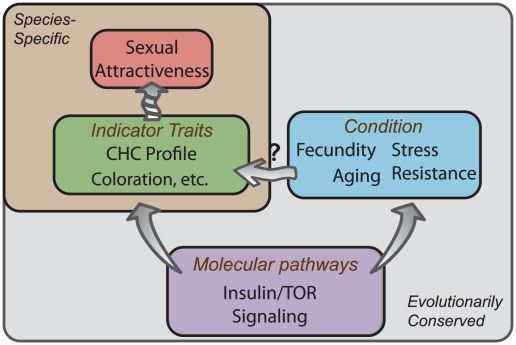
Conserved molecular pathways underlie attractive characteristics as honest indicators of condition. Knockdown of IIS and TOR signaling is known to influence an individual's condition by either potentiating or inhibiting the fitness traits such as longevity, stress resistance, and reproductive output. We find that modulation of these pathways influences sexual attractiveness, at least in part, through transcriptional regulation of genes involved in CHC production. While many attractive characteristics are species-specific (green box), a small number of conserved molecular pathways (purple box) modulate lifespan and reproduction across taxa (blue box). This convergence at the molecular level suggests that individuals may have evolved the ability to perceive conspicuous signals of such molecular activity, such as CHC profiles in flies, that accurately reflect the reproductive value of a potential mate.

Regardless of whether CHC production is a *bona fide* target of IIS, our data support a model whereby CHC profiles constitute reliable physiological indices of molecular pathways that determine fitness (Figure 5). Such indicator traits are honest, therefore, not because they are costly to produce but because their expression is tightly linked to the activity of these underlying major molecular pathways. Cheaters would therefore suffer from altering IIS to change CHC through pleiotropic effects on physiology, which would bring them out of line with existing environmental conditions and reduce individual fitness. We suggest that many sexually attractive characteristics, including those unique to individual species, may convey a universal aspect of beauty by accurately representing the molecular activity of a small number of highly conserved pathways that influence longevity and reproductive output across taxa. It will be interesting to determine whether IIS and possibly TOR signaling also impact attractiveness in other species, such as nematodes, mice, or humans, where the activities of these pathways have important health consequences.

## Materials and Methods

### Fly stocks and husbandry

Canton-S, w1118, and UAS-GFP was obtained from the Bloomington Stock Center. *chico* mutant flies and *UAS*-*dFoxo* flies were provided by M. Tatar [51] and L. Partridge [27], respectively. *chico* and their respective control flies are maintained contemporaneously in the same population and segregation of *chico* alleles is maintained by propagation of heterozygotes (normal-size, cinnabar). Segregating genotypes among sibs were identified as: ch1/+ normal-size, cinnabar; ch1/ch1, dwarf, cinnabar; +/+, normal-size, apricot [51]. The *dfoxo^w24^* strain was obtained from K. Weber [27], [52] and was subsequently backcrossed to a w^1118^ control strain for over 20 generations. This strain lacks four of five Foxo isoforms and has reduced expression of the fifth (dFoxoA). It is therefore expected to be a strongly hypomorphic allele. *UAS-Akt^RNAi^* was purchased from the VDRC stock center. *UAS-Pten/CyO* was provided by S. Leevers, and *UAS-InR* was obtained from B. Edgar [53]. TOR dominant negative (UAS-*TOR^TED^*) flies were obtained from the Bloomington Stock Center [47]. Oenocyte-less flies were created from the progeny of the cross of ‘+; *PromE(800)-Gal4, tubP-Gal80^ts^*; +’ to ‘*+*; *UAS-StingerII, UAS-hid/CyO*; +’; both strains were provided by J. Levine. *tublin5*-GeneSwitch flies were made by cloning the promoter of alphatubulin into the pSwitch2 vector.

The generation of oenocyte-less flies, which are largely devoid of CHC, followed published protocols [40]. Briefly, the progeny of the cross of “+; PromE(800)-Gal4, tubP-Gal80^ts^; +” to “+; UAS-StingerII, UAS-hid/CyO; +” were maintained at 18°C until eclosion. Following emergence, adult were kept at 25°C for at least 24 h. Then flies were subjected to three overnight heat treatments at 30°C (on days 2, 3 and 4) and left to recover for at least 24 h. GFP fluorescence was checked to confirm oenocyte ablation.

For all experiments, larvae were cultured in cornmeal-sugar-yeast “larval” media, and virgin adults were collected shortly after eclosion. For Canton-S, *chico*, *dfoxo^w24^* and *chico*; *dfoxo^w24^* double mutants (and control), flies were kept on 10% sugar/yeast (SY) food. All other mutants made by crossing *tublin5*-GeneSwitch flies to specific UAS-lines (*Akt^RNAi^, Pten, InR, dfoxo* and *TOR^TED^*) were placed into 10% SY food with RU486 (200 µM) to activate transgene expression (treatment) or with vehicle only (80% ethanol) (control) for 10–15 days before experiments. All flies were maintained at 25°C and 60% relative humidity in a 12∶12 h light∶dark cycle. Fresh food was provided every 2 or 3 days. Detailed media recipes can be found in Poon el al. [54].

### Cuticular hydrocarbon analysis

Independent procedures were applied to collect aged flies (*chico* and control) for examing CHC in GC/MS and LDI-MS. For GC/MS, a large cohort of each genotype was established by collecting virgin females into vials following eclosion. CHC samples were extracted from these cohorts every 2–3 weeks. In contrast to the GC/MS analysis, multiple, independent cohorts were established for LDI-MS measurement every 2–3 weeks, and all flies were sampled on the same day for CHC analysis.

#### Gas chromatography mass spectrometry analysis (GC/MS)

Three replicate CHC samples were prepared for each age and each genotype. For each sample, 5 flies from a single vial were placed in 100 µl of hexane. Extractions were allowed to incubate at RT for 30 min. The cuticular extract was removed and placed in a clean glass vial. The solvent was then evaporated under a chemical hood. Extracts were stored at −80°C and re-dissolved in 30 µl of heptane prior to GC/MS analysis.

The GC/MS method was followed as in a previous study [34]. The analysis was performed with a Quattromicro-GC/MS (Waters) equipped with an HP-5 column (5%-Phenyl-methylpolysiloxane column; 30 m length, 0.32 mm ID, 0.25 µm film thickness; Agilent). Ionization was achieved by electron ionization (EI) at 70 eV. One microliter of the sample was injected using a splitless injector. The helium flow was set at 1.3 ml/min. The column temperature program began at 50°C for 2 min, and increased to 300°C at a rate of 15°C/min. A quadrupole mass spectrometer was set to unit mass resolution and 3 scans/sec, from *m/z* 37 to 700. Chromatograms and mass spectra from the analysis were analyzed using MassLynx (Waters). Compounds were determined on the basis of retention time and EI mass spectra. The intensity of each compound was calculated as the area under its corresponding peak.

#### Laser desorption/ionization orthogonal time-of-flight mass spectrometry (LDI-MS)

We closely followed the method outlined in Yew *et al.* [35] for detecting CHC by LDI-MS. To account for biological variability, quantitative data were derived by averaging the signal intensities obtained by measuring 3–5 individual flies for each age or genotype. Flies were anesthetized and mounted with fine forceps onto adhesive tape (G304) attached to a glass coverslip. The coverslip was attached to a milled-out, custom-built sample plate with adhesive tabs. The integrity of the fly body remains intact during analysis in the mass spectrometer. The orthogonal mass spectrometer is equipped with an N_2_ laser emitting 3 ns long pulses at a wavelength of 337 nm and a repetition rate of 30 Hz. The laser beam spot size on the sample is ∼200 µm in diameter and has an approximately flat-top intensity profile. Ions were generated in a buffer gas environment using 2 mbar of Argon gas. For acquisition of a mass spectrum, 900 laser pulses were applied to one spot (or small area of body parts) over 30 sec. Laser fluence (light energy per pulse and area) was adjusted to values moderately above the ion detection threshold, corresponding to values between 100–200 J/m^2^. All data were acquired in positive ion mode, and mass spectra were processed using the MoverZ software (v. 2001.02.13, Genomic Solutions). Potassiated molecules formed the dominant peaks for signals corresponding to hydrocarbons in all recorded LDI-MS mass spectra. Elemental composition assignments are based on the assumption that the observed and theoretical mass values agree within +/−0.02 Da.

#### Relative quantitation

Because of compound-dependent detection efficiencies, both GC/MS and LDI-MS analyses do not allow for the determination of absolute expression levels of individual CHC compounds. Moreover, total ion signal intensities obtained by the LDI-MS analysis also depend on the exact position of the fly relative to the laser beam and by biological variability. Therefore, a normalization strategy was used to retrieve the relative changes in expression levels of individual CHC. Signal intensities of the compounds were therefore divided by the sum of the peak intensities of all identified hydrocarbons. Chromatographic peak intensities were used to normalize GC/MS data, and ion signal intensities were used for LDI-MS data.

### Behavior

#### Two-choice courtship assay

A standard courtship assay was used to test male preference (i.e., female attractiveness) between *Akt* knockdown or control females. Assays were performed under both normal and dark conditions. For each measure, two subject females for comparison were decapitated and placed on the opposite sides of a single well of a standard 24-well cell culture plate containing standard 10% SY fly medium. One 6–8 day old Canton-S virgin male was subsequently aspirated into the cell, and the duration of courtship behaviors (including orientation, wing vibration and attempted copulation) directed toward each female was recorded for 10 min. The assay was conducted in the morning and at 25°C. Behaviors were only considered to involve courtship if their duration was greater than 20 sec. To control for individual variability in total courtship behavior, male preference is presented as the percentage of time males were courting mutant females divided by the total courtship time.

#### Two-choice video analysis

To more accurately quantify the extent of preference, we also employed video recording followed by video analysis using fly tracking software. Video analysis was used to assess male choice between mutant and control females experiencing knockdown of *Akt*, overexpression of *Pten* or overexpression of *InR*. In this assay, two-choice subject females (i.e., mutant and control) were decapitated and embedded in agar 15–20 mm apart and 7–10 mm away from the side of the dish. After the agar solidified, a single, 4–8 day-old virgin Canton-S male was released in the arena and given 15–20 min to acclimate to the new environment. Video recording was then started and continued for 30 min.

Videos were recorded at 2 frames per second and converted to AVI file format, which was analyzed with our VideoFly software. The software calculates the amount of time spent by the choosing fly inside a circle of 3 mm radius centered on each decapitated subject fly. Instances where the total time spent in the two target regions was less than 50 sec (2.8% of the total time of observation) were removed from further analysis. As with our courtship assay, male preference was calculated as the percentage of time males spent in the circles centered on mutant divided by the total time spent in both circles. Detailed comparisons of the results from observed courtship assays and video assays confirmed that the latter accurately reflect male courtship behaviors as opposed to just describing proximity to females.

#### Perfuming experiments

CHC are insect cuticular waxes that are known to transfer between individuals via physical contact/“rubbing” [8], and several *Drosophila* studies have used this feature to experimentally transfer CHC between flies [40], [55], [56]. To establish that changes in the attractiveness of genetically manipulated females are mediated by CHC we conducted two types of CHC transfer experiments. Both methods involved vortexing a large number of donor flies with a smaller number or target, recipient flies. Vortexing was performed in a 2 ml glass vial for 5 min at 2/3 speed setting, immediately followed by decapitation of target females, and their placement into 2-choice videoanalysis assay. In the first set of “perfuming” experiments, 30–40 oenocyteless females devoid of CHC, were vortexed with 8 times as many Geneswitch>UAS-*InR* (RU+ or RU−) donor females, or with 6 times as many Geneswitch>UAS-*Akt^RNAi^* (RU+ or RU−) donor females. In the second, “masking”, experiment, Geneswitch>UAS-*Akt^RNAi^* (RU+ or RU−) females served as donors, and they were vortexed with 6 times as many Canton-S virgin females. These masked transgenic flies from RU+ and RU− treatments were compared in 2-choice attractiveness assay, along with non-masked flies in the control experiment.

### Quantitative real-time PCR

Total RNA was extracted from 10 virgin females at 10–15 days of age by Trizol (Invitrogen). Extracted RNA was treated with 1 U DNAse I (Invitrogen) and reverse transcribed into cDNA by Superscript III First-Strand Synthesis (Invitrogen) using oligo-dT primers. For each RNA extraction, five replicate RT-PCR reactions were performed using an ABIPrism 7000 and RT^2^ SYBR Green/Rox PCR Master Mix (SA Biosciences) with specific primers. The quantitative levels were normalized to an endogenous control *rp49*, calculated by the ΔΔCT method, and presented as fold-change of mutant to wildtype in expression levels. The results for CHC synthesis genes (*eloF*, *desat1*, *desat2* and *desatF*) were based on at least three, independent RNA extractions. The following primers were used: *desat1*F (TGCCGATTGCTTGCTTCAT), *desat1*R (TTCACCCCAGGCGTACATG), *desat2*F (GGTGGTGCTTCCAGCTAAACA), *desat2*R (GGCGATTTCCGAATTTATGG), *desatF*F (TCCGTGTGGGTGAGGGATA), *desatF*R (AGCTCGGCGCTCTTGTAGTC), *eloF*F (CCATTATTCTGCTCCACTGTACCA), *eloF*R (GTCTGTTGACCGCGCAGTT), *Rp49*F (ACTCAATGGATACTGCCAG) and *Rp49*R (CAAGGTGTCCCACTAATGCAT).

### Statistics

For GC/MS and LDI-MS data, pairwise comparisons between IIS mutants and control flies were examined by two-factor ANOVA. Statistical analysis and data presentation (see Figure 1) used CHC values after transformation to the natural log scale, where it was determined that model residuals were sufficiently normally distributed and independent of fitted values. A single potential outlier was present for each of four individual CHC. After removal and reanalysis, all four compounds retained their significance, and P-values were substantially reduced in all cases. For consistency, therefore, we report the conservative P-values from ANOVA using all data. Data from only one compound in the GC data (7-H) exhibited a significant genotype×age interaction (P = 0.004). Standard least-squares regression was used to determine the correlation between *chico* and other IIS mutants (Figure S1) and the correlation between carbon chain length and the percent change of normalized intensity in IIS mutant from control (Figure 3). It should be noted that these P values may be liberal because, without detailed knowledge of the biochemical pathways of all CHC, we can not rule out that the levels of some CHC may be correlated. *chico* data represent the genotype main effect derived using data from all ages, while data from other genotypes represent replicate measures obtained at roughly two weeks of age. Principle component analysis (PCA) on correlations followed by ANCOVA was used to visualize the effect of aging and *chico* mutation (Figure 3). PCA was done using 72 CHC samples from transgenic flies manipulated for different components of the insulin signaling pathway and their appropriate controls. PC1 was responsible for 44% of the variation and is represented by positively loading C21–26 CHC (8 CHC have factor loadings of >0.8, and another 4 CHC have factor loading of >0.6) and negatively loading 7-H (−0.680) and 7,11-ND (−0.735). PC2 explains 13% of variation and is represented by three positively loading C25 compounds, and 2MeC_28_. For both courtship assay and video analysis (Figure 2), a Wilcoxon signed rank test was applied to test the null hypothesis of no preference (no difference from 50%). For quantitative PCR, a z-test was applied to test the null hypothesis of no change in expression level. Analyses were performed using JMP 8.0.1 and R 2.13.0.

To avoid biasing results due to timing and positioning in behavioral trials (timing of decapitation and placement in choice chambers, position relative to the light source), females from different experimental treatments (RU+ and RU−) were alternated in space and time. When several replicates were perfomed, they were pooled together and significance values were determined by a permutation procedure whereby treatment labels were randomized among flies within a specific replicate. For each of 30,000 randomizations, an attractiveness value was calculated, and the 30,000 values were then pooled together to create the null distribution. One- or two-sided p-values were then determined by integrating appropriate tails of the null distribution that were more extreme than the observed attractiveness value.

## Supporting Information

Figure S1 Changes in CHC profiles caused by *chico* mutation and other IIS manipulations are highly correlated. Changes in the relative abundance of individual CHC in flies experiencing either (A) knockdown of *Akt* or (B) overexpression of *Pten* are highly positively correlated with changes observed in *chico* mutant flies. (C) Changes in CHC levels following *InR* overexpression are significantly negatively correlated with changes caused by the *chico* mutation. (D) No correlation in CHC change is observed between *chico* and overexpression of *dFoxo*. (E) Overexpression of *TOR^TED^* resulted in changes in CHC profiles that were highly correlated with those caused by mutation of *chico*. Each data point represents the difference of normalized intensity between genetically manipulated flies and their respective control in GC/MS data. *chico* effects were calculated based on the genotype main effect across all measured ages (6, 23, 37, and 48 days old), while *Pten*, *dFoxo*, and *TOR^TED^* data were based on measures obtained from two-week old flies.
(PDF)

Figure S2 Examples of CHC whose relative abundances are significantly altered by insulin signaling. Knockdown of IIS by knockout of *chico*, knockdown of *Akt*, and overexpression of *Pten* decrease the levels of 7-T, nC23, 9-P, 7,11-PD, and 7-P but increase the levels of 2-MeC_26_, 5,9-HD and 7,11-ND. In most cases, overexpression of *InR* generates changes in the opposite direction. The Y-axis presents the differences in normalized CHC intensity between control flies and flies with IIS manipulation. Data are derived from female flies by GC/MS analysis. P-values were determined by t-test: *P<0.05, **P<0.01, ***P<0.001. ND = Not determined.
(PDF)

Figure S3 RU486 has no effect on CHC profiles or organism attractiveness. (A) Individual CHC levels and CHC profiles examining the effect of RU486 alone (when administered to flies carrying the Geneswitch driver and UAS-*GFP* transgene) reveal that there are no compounds that exhibit significant differences in normalized intensity between groups of flies fed RU486 from non-RU-fed controls. Three compounds exhibited P-values less than 0.10 (9-C27:1, P = 0.053; nC25, P = 0.068; and 7-P, P = 0.081). Flies were 10–14 days of age. (B) Consistent with a general lack of RU486-based effects, there is no relationship between carbon chain length and the percentage change in individual compounds. Each data point represents the percentage change of normalized CHC abundance in flies fed RU486 compared to non-RU-fed controls for a single compound from GC/MS. (C) In multiple trials, male flies exhibited no consistent preference for females carrying the Geneswitch driver but no specific transgene on either RU486+ or RU486− food. Replicates 2 and 3 were generated alongside data presented in Figure 2B and 2C. Together, these data indicate that RU486 alone is not sufficient to generate the observed differences in the traits examined in this study. The data analyses and presentation are as described in Figure 3A for CHC compound chain length.
(PDF)

Figure S4 The effect of loss of function of *chico* on expression of genes involved in CHC synthesis is *dFoxo*-independent. The levels of mRNA for genes known to be involved in CHC synthesis (*eloF*, *desat1*, *desat2* and *desatF*) are elevated in female flies carrying a *chico* loss of function mutation (see also Figure 4). These differences largely persist in *chico*; *dfoxo^w24^*double mutant flies. Expression levels are presented as the fold-change of the mutants (*chico*, *dfoxo^w24^*, and *chico*; *dfoxo^w24^*) compared to control.
(PDF)

Table S1 The difference of normalized CHC intensity from GC/MS analysis between mutants and wild types. Table values present the difference in normalized intensity between mutants and their controls for each individual CHC as measured by GC/MS. *chico* effects were calculated based on the genotype main effect across all measured ages (6, 23, 37, and 48 days old), while *Pten*, *dFoxo*, and *TOR^TED^* data were based on measures obtained from two-week old flies. OX indicates overexpression of *Pten*, *InR* and *dFoxo*. Significance was determined by t-test *P<0.05.
(PDF)
