# Supplementary figures and images for: Insulin Signaling Mediates Sexual Attractiveness in Drosophila
Source: PLoS Genet. 2012 Apr 26;8(4):e1002684. doi: 10.1371/journal.pgen.1002684 (PMC3343104; doi:10.1371/journal.pgen.1002684)

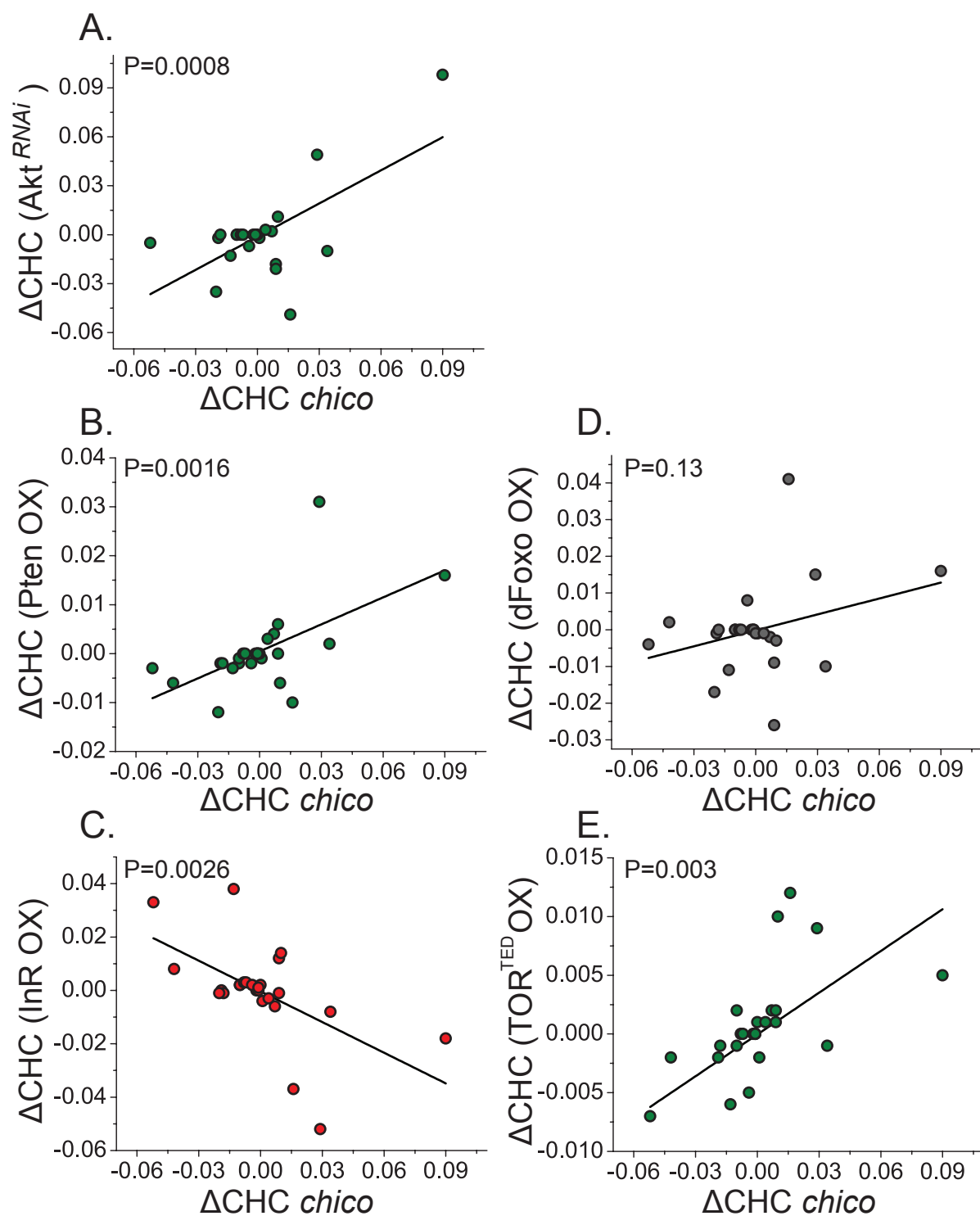

Figure S1

Supplement: Figure S1 — Changes in CHC profiles caused by chico mutation and other IIS manipulations are highly correlated. Changes in the relative abundance of individual CHC in flies experiencing either (A) knockdown of Akt or (B) overexpression of Pten are highly positively correlated with changes observed in chico mutant flies. (C) Changes in CHC levels following InR overexpression are significantly negatively correlated with changes caused by the chico mutation. (D) No correlation in CHC change is observed between chico and overexpression of dFoxo. (E) Overexpression of TORTED resulted in changes in CHC profiles that were highly correlated with those caused by mutation of chico. Each data point represents the difference of normalized intensity between genetically manipulated flies and their respective control in GC/MS data. chico effects were calculated based on the genotype main effect across all measured ages (6, 23, 37, and 48 days old), while Pten, dFoxo, and TORTED data were based on measures obtained from two-week old flies. (PDF) [file pgen.1002684.s001.pdf]

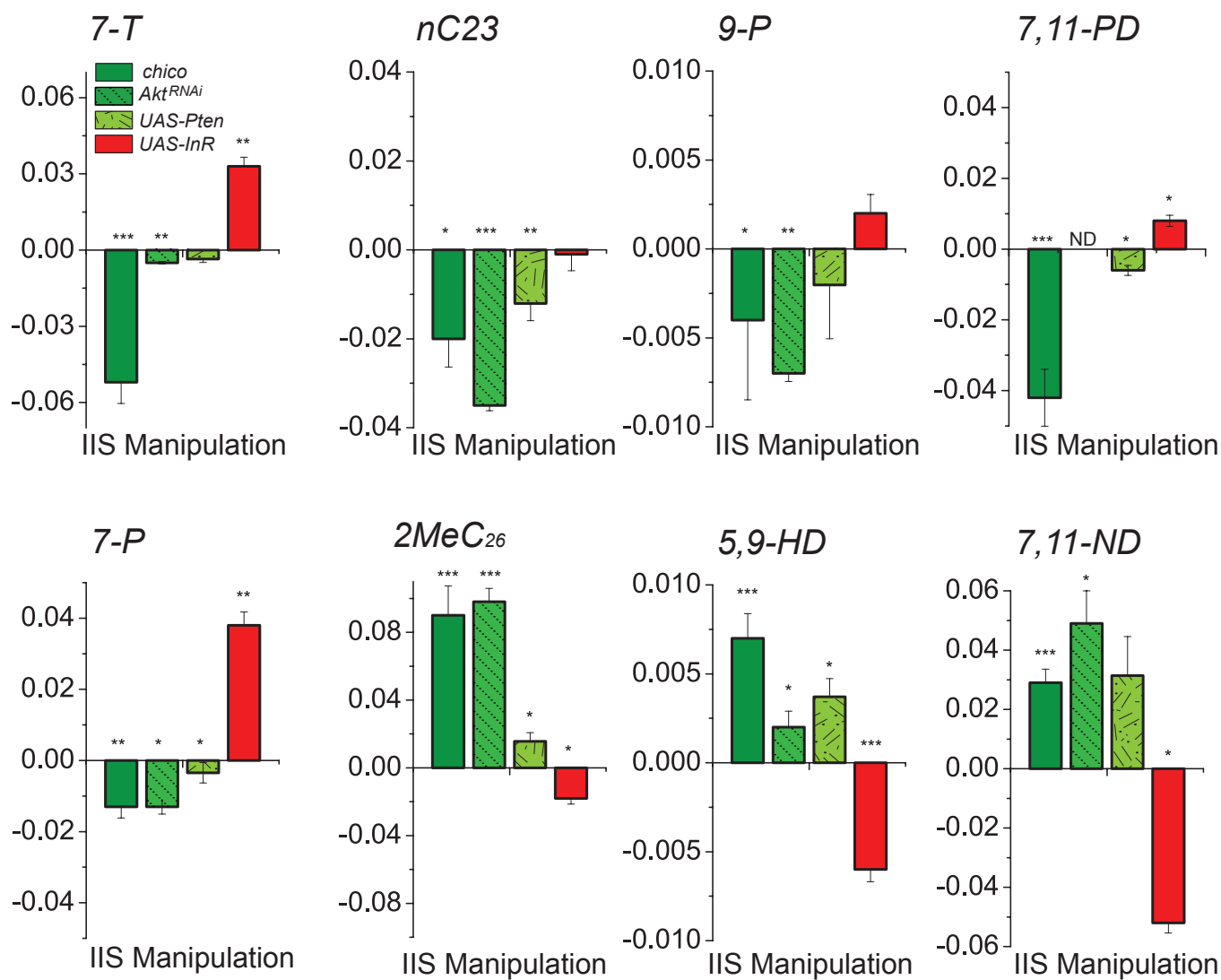

Figure S2

Supplement: Figure S2 — Examples of CHC whose relative abundances are significantly altered by insulin signaling. Knockdown of IIS by knockout of chico, knockdown of Akt, and overexpression of Pten decrease the levels of 7-T, nC23, 9-P, 7,11-PD, and 7-P but increase the levels of 2-MeC26, 5,9-HD and 7,11-ND. In most cases, overexpression of InR generates changes in the opposite direction. The Y-axis presents the differences in normalized CHC intensity between control flies and flies with IIS manipulation. Data are derived from female flies by GC/MS analysis. P-values were determined by t-test: *P<0.05, **P<0.01, ***P<0.001. ND = Not determined. (PDF) [file pgen.1002684.s002.pdf]

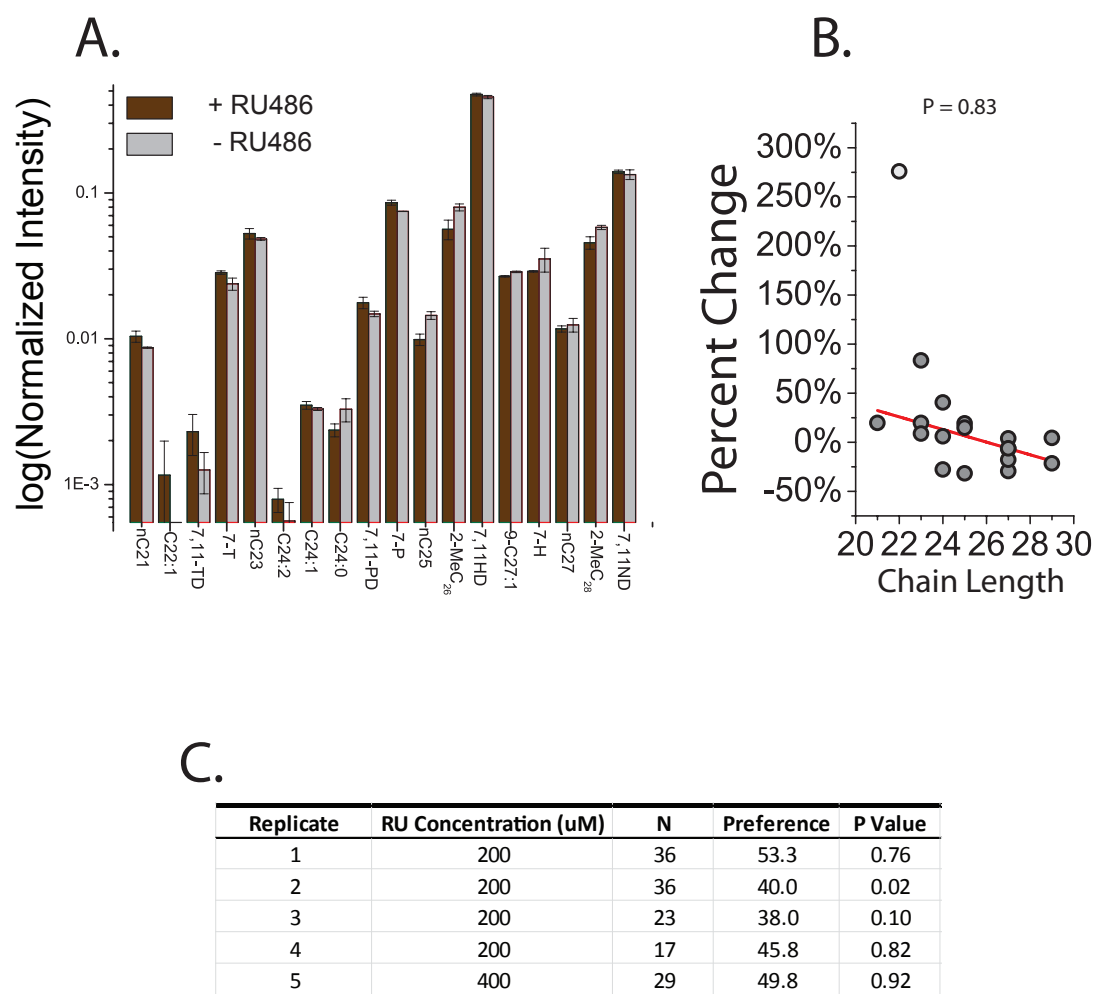

Figure S3

Supplement: Figure S3 — RU486 has no effect on CHC profiles or organism attractiveness. (A) Individual CHC levels and CHC profiles examining the effect of RU486 alone (when administered to flies carrying the Geneswitch driver and UAS-GFP transgene) reveal that there are no compounds that exhibit significant differences in normalized intensity between groups of flies fed RU486 from non-RU-fed controls. Three compounds exhibited P-values less than 0.10 (9-C27:1, P = 0.053; nC25, P = 0.068; and 7-P, P = 0.081). Flies were 10–14 days of age. (B) Consistent with a general lack of RU486-based effects, there is no relationship between carbon chain length and the percentage change in individual compounds. Each data point represents the percentage change of normalized CHC abundance in flies fed RU486 compared to non-RU-fed controls for a single compound from GC/MS. (C) In multiple trials, male flies exhibited no consistent preference for females carrying the Geneswitch driver but no specific transgene on either RU486+ or RU486− food. Replicates 2 and 3 were generated alongside data presented in Figure 2B and 2C. Together, these data indicate that RU486 alone is not sufficient to generate the observed differences in the traits examined in this study. The data analyses and presentation are as described in Figure 3A for CHC compound chain length. (PDF) [file pgen.1002684.s003.pdf]

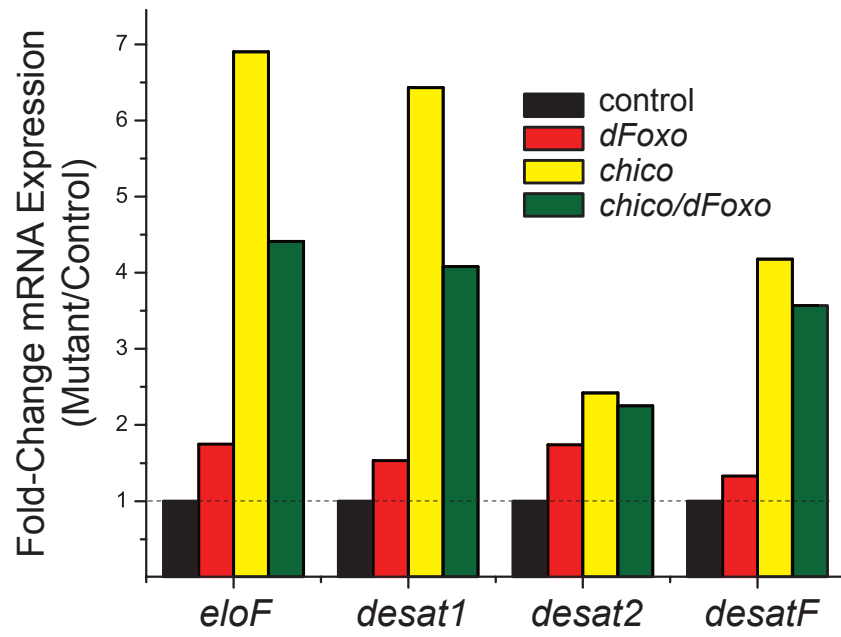

Figure S4

Supplement: Figure S4 — The effect of loss of function of chico on expression of genes involved in CHC synthesis is dFoxo-independent. The levels of mRNA for genes known to be involved in CHC synthesis (eloF, desat1, desat2 and desatF) are elevated in female flies carrying a chico loss of function mutation (see also Figure 4). These differences largely persist in chico; dfoxow24double mutant flies. Expression levels are presented as the fold-change of the mutants (chico, dfoxow24, and chico; dfoxow24) compared to control. (PDF) [file pgen.1002684.s004.pdf]
