# Supplementary material for: Insulin Signaling Mediates Sexual Attractiveness in Drosophila
Source: PLoS Genet. 2012 Apr 26;8(4):e1002684. doi: 10.1371/journal.pgen.1002684 (PMC3343104; doi:10.1371/journal.pgen.1002684)
Supplement: Table S1 — The difference of normalized CHC intensity from GC/MS analysis between mutants and wild types. Table values present the difference in normalized intensity between mutants and their controls for each individual CHC as measured by GC/MS. chico effects were calculated based on the genotype main effect across all measured ages (6, 23, 37, and 48 days old), while Pten, dFoxo, and TORTED data were based on measures obtained from two-week old flies. OX indicates overexpression of Pten, InR and dFoxo. Significance was determined by t-test *P<0.05. (PDF) [file pgen.1002684.s005.pdf]

Compound	Insulin signaling mutant (mutant - wt)				*Foxo*^{OX}	*TOR*^{TED}
	chico	*Akt*^{RNAi}	*Pten*^{OX}	*InR*^{OX}		
C21:0 (nC21)	-0.010*	0.000	-0.002	0.002	0.000	0.002
C22:1	-0.001*	0.000	0.000*	0.001*	0.000	0.000*
C22:0	-0.019*	-0.002	-0.002*	0.000	-0.001	-0.002
7,11-C23:2 (7,11-TD)	-0.018*	0.000*	-0.002*	-0.001*	0.000	-0.001*
9-C23:1	-0.010*	0.000*	-0.001*	0.002*	0.000	-0.001*
7-C23:1 (7-T)	-0.052*	-0.005*	-0.003	0.033*	-0.004*	-0.007
5-C23:1	-0.008*	0.000	0.000	0.003*	0.000	0.000
C23:0 (nC23)	-0.020*	-0.035*	-0.012*	-0.001	-0.017	-0.019*
C24:2	-0.002*	0.000*	0.000	0.000	0.000	0.000
C24:1	-0.007*	0.000	0.000*	0.003*	0.000	0.000
C24:0	0.001	-0.002*	-0.001*	-0.004*	-0.001	-0.002
9,13-C25:2	-0.001*		0.000*	0.000	0.000	0.000
7,11-C25:2 (7,11-PD)	-0.042*		-0.006*	0.008*	0.002	-0.002
9-C25:1 (9-P)	-0.004*	-0.007*	-0.002	0.002	0.008*	-0.005
7-C25:1 (7-P)	-0.013*	-0.013*	-0.003*	0.038*	-0.011*	-0.006
5-C25:1	0.000	0.000	0.000	0.002	-0.001	0.001
C25:0 (nC25)	0.034*	-0.010*	0.002	-0.008*	-0.010*	-0.001
C26:2	-0.001*	0.000	0.000	0.001		0.000
2-MeC26	0.090*	0.098*	0.016*	-0.018*	0.016	0.005
7,11-C27:2 (7,11-HD)	0.016*	-0.049*	-0.010	-0.037*	0.041	0.012
5,9-HD	0.007*	0.002*	0.004*	-0.006*	-0.002	0.002
9-C27:1	0.004*	0.003	0.003	-0.003*	-0.001	0.001
7-C27:1 (7-H)	0.009*	-0.018*	0.006*	-0.001	-0.026	0.001
C27:0 (nC27)	0.009*	-0.021*	0.000	0.012	-0.009	0.002
C28:0		0.002	0.000	0.001	0.000	0.000
2-MeC28	0.010*	0.011*	-0.006*	0.014*	-0.003	0.010
7,11-C29:2 (7,11-ND)	0.029*	0.049*	0.031	-0.052*	0.015	0.009
C29:0	-0.010*	-0.002	0.000	0.003	0.000	0.001
2-MeC30	-0.001*	-0.001	-0.008	0.006*	0.000	0.002
